# *ATM* germline variants in a young adult with chronic lymphocytic leukemia: 8 years of genomic evolution

**DOI:** 10.1038/s41408-022-00686-6

**Published:** 2022-06-07

**Authors:** Romina Royo, Laura Magnano, Julio Delgado, Sara Ruiz-Gil, Josep Ll. Gelpí, Holger Heyn, Malcom A. Taylor, Tatjana Stankovic, Xose S. Puente, Ferran Nadeu, Elías Campo

**Affiliations:** 1 Barcelona Supercomputing Center (BSC), Barcelona, Spain; 2 Institut d’Investigacions Biomèdiques August Pi i Sunyer (IDIBAPS), Barcelona, Spain; 3 Centro de Investigación Biomédica en Red de Cáncer (CIBERONC), Madrid, Spain; 4 Hospital Clínic of Barcelona, Barcelona, Spain; 5 Universitat de Barcelona, Barcelona, Spain; 6 CNAG-CRG, Centre for Genomic Regulation (CRG), Barcelona Institute of Science and Technology (BIST), Barcelona, Spain; 7 Universitat Pompeu Fabra (UPF), Barcelona, Spain; 8 Institute of Cancer and Genomic Sciences, University of Birmingham, Edgbaston, UK; 9 Departamento de Bioquímica y Biología Molecular, Instituto Universitario de Oncología, Universidad de Oviedo, Oviedo, Spain

**Keywords:** Cancer genomics, Chronic lymphocytic leukaemia, Cancer genetics

Chronic lymphocytic leukemia (CLL) is a disease commonly diagnosed in the elderly with a median age of ~70 years. However, CLL can also be detected in adolescent and young adults (AYA). According to different studies, 0.85–3.7% of patients with CLL are diagnosed in AYA and 3% of these patients had a first-degree relative with CLL [1]. Families with multiple individuals affected with CLL and other related B-cell tumors have been described with contradictory findings regarding their potential early age at diagnosis [2]. Despite these observations, our knowledge about the molecular profile and predisposing factors in AYA CLL is scarce [3, 4].

Comprehensive studies have dissected the (epi)genomic, and transcriptomic landscape of CLL [5]. Approximately 9–18% of CLL harbor del(11q) which occurs in younger patients with bulky disease and poor survival. These deletions are frequently associated with germline and acquired mutations of *ATM* [6]. Patients with the inherited disorder ataxia telangiectasia have biallelic alterations of the *ATM* gene and increased susceptibility to lymphoid malignancies [7]. Rare, protein-coding germline *ATM* variants are associated with CLL in adults [8]. However, *ATM* mutations are uncommon in familial CLL [9].

Here, we describe an 18-year-old woman diagnosed with CLL whose family history included a younger brother with B-cell acute lymphoblastic leukemia (B-ALL) and other family members carrying germline *ATM* mutations. A combination of whole-genome and single-cell characterization of this CLL at diagnosis and during the course of the disease provided an opportunity to understand the genomic profile of AYA CLL and the sequence of events driving its evolution.

An 18-year-old female was diagnosed with CLL, Binet-Rai stage AI, at another institution, in the study of a lymphocytosis detected in a routine blood test. She had a past medical history of anxiety-depressive syndrome during childhood and chronic headache, but no neurological symptoms were reported. The patient had a younger brother diagnosed with B-ALL when he was 3 years old, and was in complete remission 13 years later, and an older sister with epilepsy. Her parents were both healthy.

At the time of CLL diagnosis, the patient was asymptomatic with a normal physical exam. Her white blood cell count (WBC) was 9.08 × 10^9^/L, with 75% lymphocytes. Hemoglobin and platelet count were normal. Peripheral blood smear showed small atypical lymphocytes consistent with CLL, which phenotype was CD5^+^, CD23^+^, CD43^+^, CD200^+^, CD10^−^, CD20 and CD22 weakly positive with weak kappa light chain restriction. The fluorescence in situ hybridization (FISH) analysis for *ATM* (11q22), *D12Z3* (cen 12), *DLEU* (13q14.3), *LAMP1* (13q34), and *TP53* (17p13) were normal. One year after diagnosis, the patient received two cycles of rituximab plus fludarabine and cyclophosphamide (FCR) due to progressive disease, achieving a complete remission. The patient was then referred to our hospital. Physical examination was normal without evidence of lymphadenopathy or splenomegaly. WBC count was 2 × 10^9^/L with 10% lymphocytes, hemoglobin 117 g/L, and normal platelet count. Watchful waiting was recommended. Five years later, the CLL progressed with increased lymphocytosis, inguinal, axillary, and laterocervical lymphadenopathy (2–3 cm) and splenomegaly of 4 cm below the costal margin. At that time, the karyotype was 46,XX,del[13](q12q21)[6]/46,XX[10] and a heterozygous del(13q14.3) was detected by FISH in 92% of nuclei. FISH for *ATM*, *D12Z3*, and *TP53* were normal and no *TP53* mutations were observed. The sequence of the IGHV genes showed a clonal rearrangement of the IGHV3-21 with 100% homology to the germline, not belonging to any major stereotype subset (Supplementary Tables 1, 2). Due to CLL progression, ibrutinib 420 mg per day was started and the patient achieved a partial response. However, after 20 months, ibrutinib had to be discontinued due to the severe diarrhea and acalabrutinib 100 mg every 12 h was started. Progression of CLL was observed after 13 months of treatment and rituximab and venetoclax were initiated (Fig. 1A).

**Fig. 1:**
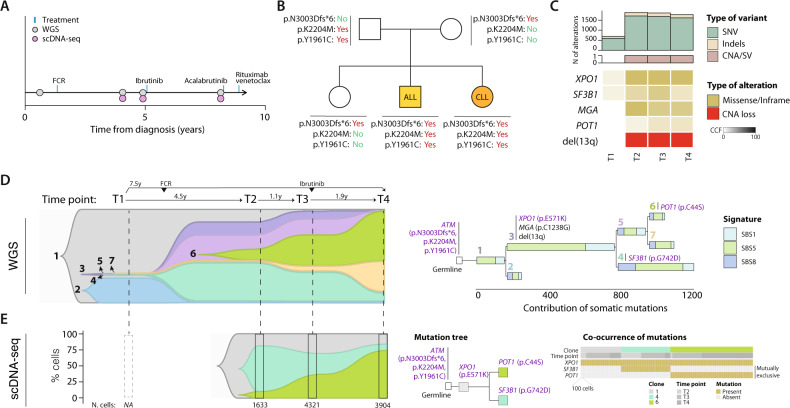
Clinical course and genomic characterization. **A** Clinical course and samples analyzed. **B** Pedigree tree of germline variants in *ATM*. The two missense variants carried by the mother and the frameshift variant from the father were inherited by the chronic lymphocytic leukemia (CLL) case studied and her brother that developed acute lymphoblastic leukemia (ALL). **C** The upper barplots show the number of mutations [single nucleotide variants (SNV) and short insertions and deletions (indels)] and copy number alterations (CNA) or structural variants (SV) at each time point. The lower oncoprint shows the driver alterations, the transparency of the color is proportional to the cancer cell fraction (CCF). **D** The fishplot [left] depicts the subclonal architecture and clonal dynamics inferred from WGS. Each vertical line represents a time point analyzed. Each subclone is painted in a different color, and its height is proportional to the CCF at each time point. The upper-right tree shows the phylogeny of the tumor cell subpopulations, the length of the branches is proportional to the number of acquired SNV, and they are colored by contribution of mutational signatures identified in CLL [right]. The clock-like signatures SBS1 and SBS5 contributed most of the mutations acquired. **E** The fishplot (left) shows the clonal dynamics measured by single-cell analysis. For each available time point, the integrated barplot shows the proportion of cells harboring each specific combination of alterations in the driver genes illustrated on the “Mutation tree” (middle). The total number of analyzed cells at each analyzed sample is shown at the bottom. The “Co-occurrence of mutations” plot (right) indicates the presence or absence of mutations in each cell. For illustrative purposes, cells have been merged in bins of 100.

The patient was included in the CLL program of the International Cancer Genome Consortium and the whole genomes of the germline and tumor sample at diagnosis were sequenced [5]. No somatically-acquired driver alterations were detected but three germline *ATM* mutations were identified, including a pathogenic 28-base frameshift deletion (p.N3003Dfs*6) and two missense single nucleotide variants (p.K2204M and p.Y1961C). Although the p.K2204M missense variant has not been identified in previous studies, the p.Y1961C has been reported in a CLL patient and its modeling showed reduced ATM kinase activity [10]. Based on this result, we studied the segregation of these mutations in the family members by Sanger sequencing. The mother harbored the frameshift deletion, while the father and the sister carried the two missense variants. Both the patient and her brother with B-ALL inherited all three variants (Fig. 1B, Supplementary Tables 3, 4). A milder ataxia telangiectasia phenotype, where the disease progresses at a slower pace, has been observed in patients with reduced levels of ATM kinase activity [11]. At time of last follow-up the two siblings (28 and 16 years old) had not developed neurological symptoms.

To better unfold the contribution of somatic alterations during the evolution of the disease, whole-genome sequencing (WGS) was performed at 3 additional time points over a period of 8 years and complemented with single-cell DNA-sequencing (Fig. 1A, Supplementary Table 1). Using a longitudinal sample-aware mutation calling pipeline that increases sensitivity, we identified 689 genome-wide and 7 non-synonymous variants in the WGS at diagnosis, increasing up to 1779 genome-wide and 18 non-synonymous at the latest sample analyzed. Among them, four mutations were found in CLL driver genes over the course of the disease: *XPO1* (p.E571K), *SF3B1* (p.G742D), *MGA* (p.C1238G), and *POT1* (p.C44S). The mutations in *XPO1* and *SF3B1* were already present at diagnosis but were missed in our previous study [5] due to their very low frequencies. After 4 years (time point 2), their clonal size expanded, and the remaining two driver mutations in *MGA* and *POT1* were detected. Regarding structural alterations, only del(13q) was clonally detected at the second time point and onwards (Fig. 1C, Supplementary Methods, Supplementary Tables 5, 8).

Somatic driver alterations were present at different allele frequencies through the disease course, suggesting an ongoing clonal evolution driving the pre- and post-treatment progression of the disease. To dissect the underlying clonal evolution, we reconstructed the subclonal evolution and explored the mutational processes active during the CLL course (Fig. 1D, Supplementary Methods, Supplementary Tables 9, 10). This analysis revealed a branching pattern of evolution in which the founding CLL clone did not carry any recognized driver alteration beyond the *ATM* germline variants. Additionally, two minor subclones were already present at diagnosis: subclone #3 carrying del(13q), *XPO1* and *MGA*, and subclone #4 which originated from subclone #3 and acquired the *SF3B1* mutation (Fig. 1D). These lineage trajectories are in line with previous literature in which *ATM* loss preceded del(13q) in a familial CLL study [12] and with a recently described combinatorial effect of *ATM* loss and *SF3B1* mutation [13]. Intriguingly, these small subclones at diagnosis expanded after treatment with FCR, that, on the other hand, reduced or eliminated the initial subclones #1 and #2, with no additional CLL drivers, suggesting that decreased competition allowed the expansion of subclones carrying potent drivers. Of note, subclone #4 carrying the *SF3B1* mutation represented the largest subpopulation of cells at relapse post-treatment with FCR (time point 2), in line with the poor prognosis of *SF3B1* mutated cases under FCR therapy [14]. Nonetheless, this subclone slightly diminished at time point 3 and was virtually eradicated at time point 4 after treatment with ibrutinib, which is in line with the higher sensitivity of *SF3B1* mutated CLL cells to BCR inhibition in vitro [13]. Additional diversification was observed in subclone #3 at time point 2 which led to the emergence of subclone #6 harboring the *POT1* mutation. This subclone expanded under ibrutinib treatment and accounted for 54% at the last time point analyzed 3 years after its detection (Fig. 1D). To confirm these evolutionary trajectories, we performed single-cell DNA-sequencing of 32 CLL driver genes and identified the reported mutations in *XPO1*, *SF3B1,* and *POT1* [note that *MGA* was not included in the commercial gene panel used]. This single-cell analysis confirmed the timing of acquisition of these driver mutations and the clonal dynamics inferred from WGS (Fig. 1E, Supplementary Methods, Supplementary Tables 11, 14).

Here we have reported the 8-year genomic evolution of a CLL diagnosed in a young patient that inherited three *ATM* variants, two of them previously reported to inactivate or reduce *ATM* activity (Supplementary Table 4) [10]. The combination of these three germline *ATM* variants predisposed to two distinct B-cell neoplasm in two siblings. These *ATM* variants represented the only recognized driver events in the founding CLL clone, suggesting that *ATM* inactivation might be a genomic factor contributing to CLL initiation. Tumor evolution and disease progression was dictated by the acquisition of secondary driver alterations, which could be detected in small subclones years before their expansion, and by different types of treatment that influenced subsequent clonal dynamics. Of note, this patient responded well to initial FCR therapy and later to ibrutinib treatment when *ATM* inactivation was accompanied by an *SF3B1* mutation, which is in line with the favorable clinical behavior of del(11q) CLL under BTK inhibitors [15]. Altogether, the lack of somatically-acquired, genetic driver alterations in the founding CLL of this patient emphasizes the need to study the germline as well as non-genetic aspects of the tumors to further understand the mechanisms leading to CLL.

## Accession number

The WGS and single-cell DNA-sequencing data have been deposited to the European Genome-phenome Archive (EGA) under the accession code EGAS00001006268.

## Supplementary information

Supplementary Materials

Supplementary Tables
