# Correction: *ATM* germline variants in a young adult with chronic lymphocytic leukemia: 8 years of genomic evolution

**DOI:** 10.1038/s41408-022-00762-x

**Published:** 2022-12-06

**Authors:** Romina Royo, Laura Magnano, Julio Delgado, Sara Ruiz-Gil, Josep Ll. Gelpí, Holger Heyn, Malcom A. Taylor, Tatjana Stankovic, Xose S. Puente, Ferran Nadeu, Elías Campo

**Affiliations:** 1 Barcelona Supercomputing Center (BSC), Barcelona, Spain; 2 Institut d’Investigacions Biomèdiques August Pi i Sunyer (IDIBAPS), Barcelona, Spain; 3 Centro de Investigación Biomédica en Red de Cáncer (CIBERONC), Madrid, Spain; 4 Hospital Clínic of Barcelona, Barcelona, Spain; 5 Universitat de Barcelona, Barcelona, Spain; 6 CNAG-CRG, Centre for Genomic Regulation (CRG), Barcelona Institute of Science and Technology (BIST), Barcelona, Spain; 7 Universitat Pompeu Fabra (UPF), Barcelona, Spain; 8 Institute of Cancer and Genomic Sciences, University of Birmingham, Edgbaston, UK; 9 Departamento de Bioquímica y Biología Molecular, Instituto Universitario de Oncología, Universidad de Oviedo, Oviedo, Spain

**Keywords:** Cancer genomics, Chronic lymphocytic leukaemia, Cancer genetics

Correction to: *Blood Cancer Journal* 10.1038/s41408-022-00686-6, published online 07 June 2022

In the sentences beginning ‘Watchful waiting...’ in this article, unnecessary texts had been inserted.

The texts should have read as follows.

Watchful waiting was recommended. Five years later, the CLL progressed with increased lymphocytosis, inguinal, axillary, and laterocervical lymphadenopathy (2–3 cm) and splenomegaly of 4 cm below the costal margin.

The original article has been corrected.

