# Supplementary material for: ATM germline variants in a young adult with chronic lymphocytic leukemia: 8 years of genomic evolution
Source: Blood Cancer J. 2022 Jun 7;12(6):90. doi: 10.1038/s41408-022-00686-6 (PMC9174185; doi:10.1038/s41408-022-00686-6)
Supplement: Supplementary file 1 — Supplementary Materials [file 41408_2022_686_MOESM1_ESM.pdf]

Supplementary Information

ATM germline variants in a young adult with chronic lymphocytic leukemia: 8 years of genomic evolution

Royo, Magnano et al.

Supplementary Methods

Sample collection

Written informed consent was obtained and the study was approved by the Hospital Clínic of Barcelona Ethics Committee. Tumor samples were obtained from fresh or cryopreserved mononuclear cells. Purification was done using a cocktail of magnetically labeled antibodies (AutoMACS, Miltenyi Biotec) (1). The germ line sample was obtained from the non-tumoral purified cells. Appropriate Qiagen kits were used to extract the DNA following manufacturer's recommendations. DNA quality and quantity were assayed by SYBR-green staining on agarose gels and quantified using a Qubit dsDNA HS assay (Invitrogen).

Identification of germline *ATM* mutations by Sanger sequencing

Amplification of the fragments of interest by PCR was performed using the Taq PCR Master Mix Kit (Qiagen) following manufacturer's recommendations using 25ng of input DNA in a final reaction volume of 25µl. The sequence of the primers used can be found in Supplementary Table 3. PCR products were cleaned using ExoSAP-IT (USB) and sequenced using ABI Prism BigDye terminator (Applied Biosystems). Sequencing reactions were run on an ABI-3730 automated sequencer (Applied Biosystems). All sequences were visually examined with the Mutation Surveyor® software (SoftGenetics).

Whole genome sequencing

Whole-genome sequencing (WGS) was performed for all samples. Two samples were included in our previous ICGC-CLL study (1). New library preparation for paired-end WGS was performed using the TruSeq DNA PCR-Free kit (Illumina) or the TruSeq DNA Nano protocol (Illumina) based on the available material following manufacturer's recommendations, and sequenced on a HiSeq X Ten (2x151 bp) or NovaSeq 6000 (2x151 bp)

instrument (Illumina) aiming at a mean coverage of 30x. Primary data analysis, image analysis, base calling, and quality scoring of the run were processed using the manufacturer's software. A sample-based summary can be found in Supplementary Table 1.

WGS analyses

Alignment and quality control: Quality control metrics of FASTQ files were extracted using FastQC (www.bioinformatics.babraham.ac.uk/projects/fastqc, v0.11.5). Raw reads were mapped onto the human reference genome (GRCh37) using BWA-mem algorithm (v0.7.15) (2). Biobambam2 (https://gitlab.com/german.tischler/biobambam2, v2.0.65) was used to sort and index the BAM files, and to flag optical or PCR duplicates. Quality control metrics of the BAM files were obtained using Picard (https://broadinstitute.github.io/picard, v2.10.2) (Supplementary Table 1).

Single nucleotide variants (SNV): Somatic SNV were identified using a multi-caller approach of 4 variant callers: Sidrón (1), CaVEMan (cgpCaVEManWrapper, v1.12.0) (3), Mutect2 (GATK v4.0.2.0) (4), and MuSE (v1.0 rc) (5). We applied caller-specific filters to remove low quality variants identified by CaVEMan and Mutect2. Variants detected by CaVEMan with CLPM > 0 and ASMD values <90, <120 or <140 for sequencing read lengths of 100, 125, or 150 base pairs, respectively, were excluded. Variants called by Mutect2 with MMQ < 60 were eliminated. Mutations detected by at least two algorithms were kept for downstream analyses.

To increase the sensitivity of our variant calling, each SNV called in at least one sample was searched in the other samples, if the variant was not initially found, and it was recovered if at least one read with the mutation was found in the BAM file using alleleCounter (v4.0.0, https://github.com/cancerit/alleleCount). Only high-quality reads and bases were considered (mapping quality >= 35, base quality >= 20).

Small insertions and deletions (indels): Indels were called using Pindel (cgpPindel, v2.2.3) (6,7), Platypus (v0.8.1) (8), SvABA (v7.0.2) (9), and Mutect2. The following caller-specific filters were applied: variants with MMQ<60, MQ<60, and MAPQ<60 for Mutect2, Platypus, and SvABA, respectively, were removed. Only indels identified by at least two algorithms were retained for downstream analyses. Indels identified in at least one time-point were added in the other sequential samples if any of the algorithms detected the alteration, regardless of its filters.

Copy number alterations (CNA): CNA were called using Battenberg (cgpBattenberg, v3.2.2) (10) and ASCAT (ascatNgs, v4.1.0) (11). A consensus between the two callers was determined by visual inspection of the results. We confirmed the results obtained using Genome-wide Human SNP Array 6.0 (Thermo Fisher Scientific) available for the first simple analyzed (1). Tumor purities were obtained from Battenberg and were double checked (and adjusted if needed) based on the distribution of the variant allele frequency of the clonal SNV (Supplementary Table 1).

Structural variants (SV): SV were detected using BRASS (v6.0.5) (12), SvABA, and DELLY2 (v0.8.1) (13). We filtered out variants called by BRASS with MAPQ<90, and those with MAPQ<60 for SvABA or DELLY2. Finally, SV identified by at least two programs and passing caller-specific filters for at least one program were kept. All SV were visually inspected using the Integrative Genomic Viewer (IGV) (14). Similar to SNV and indels, we recovered SV identified in any of the samples if they were detected by any program disregarding all filters and/or if they were seen by visual inspection using IGV.

Variant annotations and driver alterations: SNV and indels were annotated with snpEff/snpSift (v4.3t) using RefSeq (GRCh37.p13.RefSeq) (15). We compiled a catalogue of

genes considered as drivers in CLL (1,16) and annotated SNV, indel, CNA and SV disrupting these genes as drivers.

Immunoglobulin gene rearrangements, stereotypy, and IGHV mutational status: IgCaller (17) was used to analyze immunoglobulin gene rearrangements (heavy and light chain rearrangements as well as class switch recombination) from WGS. The sequences obtained from IgCaller were used as input of Curated sequences were used as input of IMGT/V-QUEST (18) to annotate the genes, functionality and IGHV mutational status based on current guidelines (19). The ARResT/AssignSubsets online tool (20) was used to analyze stereotypy.

Mutational signatures: SNV were used to identify the mutational processes active during the course of the disease. SNV were classified into 96 substitution classes considering the base substitution and their 5' and 3' flanking bases. COSMIC mutational signatures (v3) known to be found in CLL were considered (SBS1, SBS5, SBS8 and SBS9) (1,21,22). We measured their contribution using a fitting approach (MutationalPatterns, v1.12.0) and iteratively removing the less contributing signature if removal of the signature decreased the cosine similarity between the original and reconstructed 96-profile less than 0.01, as previously described (22).

Subclonal architecture and clonal evolution: SNV were used to assess the subclonal architecture and evolution of the tumor. SNV were clustered using a Bayesian method (10,23–25). First, a Markov chain Monte Carlo (MCMC) sampler for a Dirichlet process mixture model was used to infer putative subclones (assignment of mutations to subclones, and estimation of the subclone frequencies in each sample) from the SNV read counts, copy number states, and tumor purities. The MCMC sampler was run for 10000 iterations, discarding the first 5000. Clusters with less than 50 mutations were excluded. The phylogenetic tree of the subclones was identified following the "pigeonhole principle" (25), allowing a tolerated error

of 0.001. Clusters not assigned in the reconstructed tree were not considered. The length of each tree branch in the tree is proportional to the number of mutations assigned to the corresponding subclone. TimeScape R package (v1.6.0) was used to plot the fish plots.

Single-cell DNA-seq (scDNA-seq)

Sample preparation: scDNA-seq was performed for 3 different time points on a commercial gene panel (Tapestri single-cell DNA CLL panel from Mission Bio) covering 32 CLL driver genes, using the Tapestri Platform from Mission Bio. Sample and library preparation were performed following manufacturer's recommendations. Sequencing of all libraries was carried out on an Illumina NovaSeq 6000 S1 sequencer to obtain approximately 1300 reads/cell.

Data analysis: The Tapestri Pipeline (V1, Mission bio) was used to analyze the data. In short, adaptor sequences were trimmed, reads where aligned to the reference genome (hg19) using BWA, barcodes were corrected and reads were assigned to the corresponding cell barcode, and genotype calling was performed using the Genome Analysis Tooklit (GATK, v.37). Tapestri Insights (v2.2, Mission Bio) was used to analyze the output files (loom format) altogether. Genotypes with quality <30, read depth <10, or allele frequency <20% were marked as missing. Variants genotyped in less than 50% of the cells or mutated in less than 1% of the cells were not considered. Cells with less than 50% of genotypes present were removed. After applying all these filters, a mean of 5948 cells per sample was obtained. Variants detected in bulk WGS were included as a white-list on Tapestri Insights. Variants at low-frequency (1-10% of cells) in all scDNA-seq samples and not present in COSMIC were black-listed to remove potential artifacts from library preparation and/or sequencing. Only coding and splice site mutations (SNV and indels) were analyzed. Genotypes of the detected mutations were exported and used as input of ∞SCITE (https://github.com/cbg-ethz/infSCITE) (26), encoded

as follows: zero for wild type, one and two for heterozygous and homozygous mutation, respectively, and three for missing genotypes. ∞SCITE was used to infer the mutation tree and assign cells into subclones. Cells assigned to more than one subclone or genotyped as wild-type for all mutations were not considered. As previously described (27), ∞SCITE was run using a global sequencing error rate (false positive rate) of 1%, following Mission Bio's recommendation, using an estimated rate of non-mutated sites identified as homozygous mutations of 0%, and an estimated rate of allele dropout rate (false negative rate) specific of each sample. Germline single-nucleotide polymorphisms in gnomAD with a population frequency >1% and identified as mutated in at least 75% of cells with a variant allele frequency per read count between 47% and 53%, were used to estimate the rate of mutated allele and normal allele dropouts.

Supplementary Tables

Supplementary Tables are placed in the Supplementary Tables Excel file.

Supplementary Table 1: Associated metadata of WGS samples

Supplementary Table 2: Immunoglobulin gene rearrangements determined by IgCaller

Supplementary Table 3: Primers used for Sanger sequencing of *ATM*

Supplementary Table 4: *ATM* germline variants

Supplementary Table 5: Somatic mutations (SNV and indels) identified in WGS

Supplementary Table 6: Coding mutations (SNV and indels) identified in WGS

Supplementary Table 7: Copy number alterations identified in WGS

Supplementary Table 8: Structural variants identified in WGS

Supplementary Table 9: Subclonal reconstruction from WGS. Clusters identified and its abundance in each time point.

Supplementary Table 10: Mutational signatures analysis. Contribution of CLL mutational processes to each cluster (identified in the subclonal reconstruction)

Supplementary Table 11: Single-cell DNA-seq samples, metadata and genes studied

Supplementary Table 12: Single-cell DNA-seq mutations identified from Tapestri Insights

Supplementary Table 13: Single-cell DNA-seq allele dropout and doublet rates

Supplementary Table 14: Single-cell DNA-seq. Count matrices (based on infSCITE)

Supplementary References

1. Puente XS, Beà S, Valdés-Mas R, Villamor N, Gutiérrez-Abril J, Martín-Subero JI, et al. Non-coding recurrent mutations in chronic lymphocytic leukaemia. *Nature*. 2015;526(7574): 519–524.
2. Li H, Durbin R. Fast and accurate short read alignment with Burrows-Wheeler transform. *Bioinformatics*. 2009;25(14): 1754–1760.
3. Jones D, Raine KM, Davies H, Tarpey PS, Butler AP, Teague JW, et al. cgpCaVEManWrapper: Simple Execution of CaVEMan in Order to Detect Somatic Single Nucleotide Variants in NGS Data. *Current Protocols in Bioinformatics*. 2016;56(1): 15.10.1-15.10.18.
4. McKenna A, Hanna M, Banks E, Sivachenko A, Cibulskis K, Kernytsky A, et al. The genome analysis toolkit: A MapReduce framework for analyzing next-generation DNA sequencing data. *Genome Research*. 2010;20(9): 1297–1303.
5. Fan Y, Xi L, Hughes DST, Zhang J, Zhang J, Futreal PA, et al. MuSE: accounting for tumor heterogeneity using a sample-specific error model improves sensitivity and specificity in mutation calling from sequencing data. *Genome Biology*. 2016;17(1): 178.
6. Raine KM, Hinton J, Butler AP, Teague JW, Davies H, Tarpey P, et al. cgpPindel: Identifying Somatically Acquired Insertion and Deletion Events from Paired End Sequencing. *Current protocols in bioinformatics*. 2015;52: 15.7.1-12.
7. Ye K, Schulz MH, Long Q, Apweiler R, Ning Z. Pindel: a pattern growth approach to detect break points of large deletions and medium sized insertions from paired-end short reads. *Bioinformatics*. 2009;25(21): 2865–2871.
8. Rimmer A, Phan H, Mathieson I, Iqbal Z, Twigg SRF, Wilkie AOM, et al. Integrating mapping-, assembly- and haplotype-based approaches for calling variants in clinical sequencing applications. *Nature Genetics*. 2014;46(8): 912–918.

9. Wala JA, Bandopadhayay P, Greenwald NF, O'Rourke R, Sharpe T, Stewart C, et al. SvABA: genome-wide detection of structural variants and indels by local assembly. *Genome research*. 2018;28(4): 581–591.

10. Nik-Zainal S, Van Loo P, Wedge DC, Alexandrov LB, Greenman CD, Lau KW, et al. The life history of 21 breast cancers. *Cell*. 2012;149(5): 994–1007.

11. Raine KM, Van Loo P, Wedge DC, Jones D, Menzies A, Butler AP, et al. ascatNgs: Identifying Somatically Acquired Copy-Number Alterations from Whole-Genome Sequencing Data. *Current protocols in bioinformatics*. 2016;56(1): 15.9.1-15.9.17.

12. Nik-Zainal S, Davies H, Staaf J, Ramakrishna M, Glodzik D, Zou X, et al. Landscape of somatic mutations in 560 breast cancer whole-genome sequences. *Nature*. 2016;534(7605): 47–54.

13. Rausch T, Zichner T, Schlattl A, Stutz AM, Benes V, Korbel JO. DELLY: structural variant discovery by integrated paired-end and split-read analysis. *Bioinformatics*. 2012;28(18): i333–i339.

14. Robinson JT, Thorvaldsdóttir H, Winckler W, Guttman M, Lander ES, Getz G, et al. Integrative genomics viewer. *Nature Biotechnology*. 2011;29(1): 24–26.

15. Cingolani P, Patel VM, Coon M, Nguyen T, Land SJ, Ruden DM, et al. Using Drosophila melanogaster as a Model for Genotoxic Chemical Mutational Studies with a New Program, SnpSift. *Frontiers in Genetics*. 2012;3: 35.

16. Landau DA, Tausch E, Taylor-Weiner AN, Stewart C, Reiter JG, Bahlo J, et al. Mutations driving CLL and their evolution in progression and relapse. *Nature*. 2015;526(7574): 525–530.

17. Nadeu F, Mas-de-les-Valls R, Navarro A, Royo R, Martín S, Villamor N, et al. IgCaller for reconstructing immunoglobulin gene rearrangements and oncogenic translocations from whole-genome sequencing in lymphoid neoplasms. *Nature Communications*.

2020;11(1): 3390.

18. Brochet X, Lefranc MP, Giudicelli V. IMGT/V-QUEST: the highly customized and integrated system for IG and TR standardized V-J and V-D-J sequence analysis. *Nucleic Acids Research*. 2008;36(Web Server): W503–W508.

19. Rosenquist R, Ghia P, Hadzidimitriou A, Sutton LA, Agathangelidis A, Baliakas P, et al. Immunoglobulin gene sequence analysis in chronic lymphocytic leukemia: updated ERIC recommendations. *Leukemia 2017 31:7*. 2017;31(7): 1477–1481.

20. Bystry V, Agathangelidis A, Bikos V, Sutton LA, Baliakas P, Hadzidimitriou A, et al. ARResT/AssignSubsets: a novel application for robust subclassification of chronic lymphocytic leukemia based on B cell receptor IG stereotypy. 2015;31(23).

21. Kasar S, Kim J, Improgo R, Tiao G, Polak P, Haradhvala N, et al. Whole-genome sequencing reveals activation-induced cytidine deaminase signatures during indolent chronic lymphocytic leukaemia evolution. *Nature Communications*. 2015;6(1): 8866.

22. Alexandrov LB, Kim J, Haradhvala NJ, Huang MN, Tian Ng AW, Wu Y, et al. The repertoire of mutational signatures in human cancer. *Nature*. 2020;578(7793): 94–101.

23. Bolli N, Avet-Loiseau H, Wedge DC, Van Loo P, Alexandrov LB, Martincorena I, et al. Heterogeneity of genomic evolution and mutational profiles in multiple myeloma. *Nature Communications*. 2014;5(1): 2997.

24. Dentro SC, Wedge DC, Van Loo P. Principles of Reconstructing the Subclonal Architecture of Cancers. *Cold Spring Harbor Perspectives in Medicine*. 2017;7(8): a026625.

25. Maura F, Bolli N, Angelopoulos N, Dawson KJ, Leongamornlert D, Martincorena I, et al. Genomic landscape and chronological reconstruction of driver events in multiple myeloma. *Nature Communications*. 2019;10(1): 3835.

26. Kuipers J, Jahn K, Raphael BJ, Beerenwinkel N. Single-cell sequencing data reveal

widespread recurrence and loss of mutational hits in the life histories of tumors. *Genome Research*. 2017;27(11): 1885–1894.

27. Morita K, Wang F, Jahn K, Hu T, Tanaka T, Sasaki Y, et al. Clonal evolution of acute myeloid leukemia revealed by high-throughput single-cell genomics. *Nature Communications*. 2020;11(1): 5327.
